# A Code of Rules for the Prevention of Infectious and Contagious Diseases in Schools

**Published:** 1892-03

**Authors:** 


*A Code of Rules for the Prevention of Infectious and Contagious Diseases in Schools.* Third and Revised Edition. Pp. 35. London: J. & A. Churchill. 1891.

The issue of a third edition of this very valuable *Code*, drawn up by the Medical Officers of Schools Association, shows that the two previous editions have been deservedly appreciated. The alterations, though slight, tend to simplicity and usefulness, and the new edition of this handbook will continue to form an indispensable guide to schoolmasters, medical attendants, and parents.

